# Synapsable quadruplex-mediated fibers

**DOI:** 10.1186/1556-276X-8-210

**Published:** 2013-05-03

**Authors:** Miguel Angel Mendez, Veronika A Szalai

**Affiliations:** 1 Department of Chemistry and Biochemistry, University of Maryland, Baltimore County, 1000 Hilltop Drive, Baltimore, MD, 21250, USA; 2 Universidad San Francisco de Quito, Vía Interoceánica Km 2 1/2, Cumbayá, Quito, 17-1200-84, Ecuador; 3 Center for Nanoscale Science and Technology, National Institute of Standards and Technology, 100 Bureau Drive, Gaithersburg, MD, 20899-6204, USA

**Keywords:** Guanine quartet, Guanine quadruplex, Atomic force microscopy, Nanowires, Nanofibers, DNA nanomaterials, Synapsable quadruplex

## Abstract

We have fabricated a DNA-based nanofiber created by self-assembly of guanine quadruplex (Hoogsteen base pairing) and double-stranded DNA (Watson-Crick base pairing). When duplexes containing a long stretch of contiguous guanines and single-stranded overhangs are incubated in potassium-containing buffer, the preformed duplexes create high molecular weight species that contain quadruplexes. In addition to observation of these larger species by gel electrophoresis, solutions were analyzed by atomic force microscopy to reveal nanofibers. Analysis of the atomic force microscopy images indicates that fibers form with lengths ranging from 250 to 2,000 nm and heights from 0.45 to 4.0 nm. This work is a first step toward the creation of new structurally heterogeneous (quadruplex/duplex), yet controllable, DNA-based materials exhibiting novel properties suitable for a diverse array of nanotechnology applications.

## Background

Programmable self-assembly from deoxyribonucleic acid (DNA) building blocks has led to a myriad of nanoscale structures, including 3D architectures [1-8]. At the core, construction of ever more complicated and elegant DNA nanoshapes relies on the self-recognition properties of DNA. In DNA-based wires, tiles (double or triple crossover) [8-11], and DNA origami structures, canonical Watson-Crick base pairing drives and stabilizes formation of the desired structure. Non-canonical base pairing schemes are not typically exploited to create novel DNA-based materials [12], even though such interactions are in the lexicon of nucleic acid self-interactions observed in biological systems [13-23].

Several years ago, Watson-Crick self-recognition was combined with non-canonical base pairing to create ‘synapsable’ DNA [24]. Synapsable DNA is fashioned from two duplex DNA precursors that connect to form a four-stranded DNA unit with blunt ends. Each DNA strand in the unit created originally by Sen's group contains an internal run of eight guanines, which creates a region of guanine-guanine mismatches in the duplex precursor. Introduction of potassium ions induces the guanine-rich tracts in the duplex precursors to Hoogsteen base pair, creating a DNA element called a guanine quartet. In the final structure, the central six guanines are involved in creating the guanine quartets [24], and four duplex ‘tails,’ two at each end, project from the quadruplex core.

In addition to the Hoogsteen base pairing in synapsable DNA mimicking interactions and structures found in biology [13,15,19,20,25], synapsable DNA also has been suggested to be an attractive tool for nanofabrication [1,26] although there are no reports of specific examples utilizing synapsable DNA in such a capacity. For the first time, we report the assembly of synapsable DNA-based nanofibers that constitute a novel DNA molecular manufacturing element. Our structure is likely stiffer than canonical DNA-based structures, which potentially improves its ease of use in patterning and other nanotechnology applications. Further, our unique strategy is expected to create DNA building blocks with a broad temperature response range that can be modulated additionally by sequence control. Finally, our novel design permits future integration with other established and emerging programmable self-assembly methods such as DNA origami or tiles to create new multi-functional nanomaterials.

## Methods

Certain commercial entities, equipment, or materials may be identified in this document in order to describe an experimental procedure or concept adequately. Such identification is not intended to imply recommendation or endorsement by the National Institute of Standards and Technology, nor is it intended to imply that the entities, materials, or equipment are necessarily the best available for the purpose.

All DNA oligonucleotides were purchased from Midland Oligos (Midland, TX, USA). DNA was resuspended in purified water with a total organic content of less than 3.4 × 10^−5^ kg m^−3^ (34 μg/L) and a resistivity of 18.2 MΩ·cm. DNA was ethanol-precipitated using a slightly modified version of a previously reported protocol and resuspended in purified water [27]. Tetramethylammonium chloride (TMACl), ammonium persulfate, mercaptoethanol, MgCl_2_, KCl, tris(hydroxymethyl) aminomethane (Tris), boric acid, and *N*-methylmesoporphyrin IX were biochemical grade or equivalent reagents purchased from commercial suppliers. To separate and isolate DNA in some cases, microcentrifugal filter units (3,000 or 10,000 molecular weight cutoff) and hydrophilic polyvinylidene fluoride filters (0.45-μm pore size) were used. A solution of a mixture of 19 equivalents of acrylamide to 1 equivalent bisacrylamide with an acrylamide mass fraction of 40% was used for gel electrophoresis. Three types of buffer were used and are given here and listed in Table S1 in Additional file 1: 0.01 KMgTB, which is 1.0 × 10^−2^ mol/L (10 mM) KCl, 1.0 × 10^−3^ mol/L (1.0 mM) MgCl_2_, 0.05 mol/L (50 mM) Tris-borate, pH 8.0; 0.01 TMgTB, which is 1.0 × 10^−2^ mol/L (10 mM) TMACl, 1.0 × 10^−3^ mol/L (1.0 mM) MgCl_2_, 0.05 mol/L (50 mM) Tris-borate, pH 8.0; and 1 KMgTB, which is 1.0 mol/L (1 M) KCl, 1.0 × 10^−3^ mol/L (1.0 mM) MgCl_2_, 0.05 mol/L (50 mM) Tris-borate, pH 8.0. A silicon wafer substrate for atomic force microscopy was obtained from Silicon Valley Microelectronics, Inc. (Santa Clara, CA, USA). Sybr Green I Nucleic Acid Gel Stain 10 000 X was purchased from Lonza (Rockland, MA, USA).

### Standard DNA handling and purification

Oligonucleotide sequence information is in Table 1. Synthetic oligonucleotide pellets resuspended in water were ethanol-precipitated using 2.5 mol/L (2.5 M) TMACl. Typically, an equal volume of 2.5 mol/L (2.5 M) TMACl and oligonucleotide (typically 1 × 10^−3^ mol/L to 3 × 10^−3^ mol/L (1 mM to 3 mM)) in water were combined and vortexed. A volume of ethanol/water with a volume fraction of 95% ethanol (2.5 times the initial sample volume) was added, and the sample was stored at −13°C for 1 h or −80°C for 30 min. Samples were centrifuged for 90 to 100 min at 14,000 ×*g*. The ethanol supernatant was removed using a pipette, and the pellet was resuspended in purified water. Extinction coefficients for the single-stranded oligonucleotides were calculated by the nearest neighbor method and are included in Table 1[28]. The strand concentration was determined spectrophotometrically. Comparisons of experimentally measured spectra and spectra predicted using nearest neighbor-derived extinction coefficients [29] generate overall root mean square deviations of 0.013 for single-stranded DNA.

**Table 1:** Oligonucleotide sequences

**Name**	**Length**	**5′→3′ sequence**	(1) $\varepsilon_{260}^{a}$ **(L mol**^**−1**^**m**^**−1**^**)**	***ϵ***_**260**_
			**(L mol**^**−1**^**m**^**−1**^**)**	**(mM**^**−1**^**cm**^**−1**^**)**
C1A	39	ACAGTAGAGATGCTGCTGATTCGTTCATGTGCTTCAAGC	3.732 × 10^7^	373.2
C1B		TGTCATCTCTACGACGACTAAGCAAGTACACGAAGTTCG	3.769 × 10^7^	376.9
SQ1A	39	CAGTAGAGATGCTGCTGAGGGGGGGGTGTGCTTCAAGCG	3.799 × 10^7^	379.9
SQ1B		CTCTACGACGACTGGGGGGGGACACGAAGTTCGCTACTG	3.732 × 10^7^	373.2
C2	29	TCTACGACGACTGGGGGGGGACACGAAGT	2.856 × 10^7^	285.6

The G-box region in each sequence is underlined. ^a^Extinction coefficients for single-stranded oligonucleotide in SI units.

Double-stranded DNA was purified by native polyacrylamide gel electrophoresis (PAGE) in TMACl prior to use in assembling larger structures. Complementary single-stranded DNA sequences were hybridized in 0.01 TMgTB by heating to 90°C for 10 min followed by slow cooling to 25°C. TMACl inhibits guanine quadruplex formation [30]. Duplex DNA was stored at 4°C prior to further purification by native PAGE. In most cases, duplex DNA precursor was prepared immediately before gel electrophoresis. Duplex DNA requiring storage for longer than 12 h prior to electrophoresis was stored at −17°C or −80°C. Duplex DNA was purified by native PAGE (acrylamide mass fraction of 12%) run at 250 to 300 V. The electrophoresis running buffer was 0.01 TMgTB. All solutions containing TB were prepared from a TB stock solution consisting of 0.5 mol/L (0.5 M) Tris and 0.5 mol/L (0.5 M) boric acid at pH 8.0. The DNA in the gel was visualized by UV shadowing, and the gel was imaged using a digital camera. Duplex DNA was excised from the gel and recovered following standard procedures [31]. DNA was either isolated and concentrated in 0.05 mol/L (50 mM) TMACl using microfuge filtration devices (10,000 molecular weight cutoff) or ethanol-precipitated using 2.5 M TMACl as described above and resuspended in 0.01 TMgTB buffer.

### Non-denaturing PAGE of synapsable G-quadruplexes

Duplex precursors were incubated in high potassium ion-containing buffers to form quadruplexes. Control samples of the homoquadruplexes formed by SQ1A, SQ1B, or C2 were prepared by heating a single-stranded oligonucleotide to 95°C in 1 KMgTB buffer for 10 min followed by slow cooling to room temperature. For *N*-methylmesoporphyrin IX (NMM)-staining experiments, samples were incubated with NMM for at least 30 min at room temperature prior to gel loading.

Non-denaturing PAGE for gels with an acrylamide mass fraction of 15% was performed at 4°C at 300 V; gels containing an acrylamide mass fraction of 12% were run at 4°C and 250 V. The electrophoresis running buffer was either 0.01 TMgTB buffer or 0.01 KMgTB buffer. Gels were UV-shadowed, imaged by UV transillumination, or stained with Sybr Green I dye by soaking the gels for 10 to 20 min. All gels were wrapped in plastic wrap prior to imaging. UV shadowing was accomplished using a handheld UV lamp and standard digital imaging device. Transillumination to visualize NMM fluorescence was performed using a standard UV transilluminator device equipped with an ethidium bromide photographic filter. Images were processed (background subtraction, contrast adjustment) using ImageJ software. Sybr Green I-stained gels were scanned on a laser-based fluorescence imaging device and analyzed using the instrument-supplied software.

### Atomic force microscopy

For the preparation of atomic force microscopy (AFM) substrates, small squares of silicon wafer were washed at 65°C for 30 min in a cleaning solution (piranha) made of three parts sulfuric acid to one part H_2_O_2_ in H_2_O (H_2_O_2_ mass fraction of 30%) followed by rinsing three times with purified water. Cleaned silicon wafers were stored under purified water. Immediately prior to use, cleaned silicon wafer substrate squares were dried under a stream of nitrogen gas. One drop of 2 mol/L (2 M) MgCl_2_ in water (enough to cover the surface) was dropped on the silicon wafer. The substrate was washed extensively with purified water until cloudy spots were no longer visible on the surface. The wafer was then dried under a stream of nitrogen. The washing and drying process was repeated twice. At this point, 2 μL of the sample was applied to the surface and allowed to dry for 5 min. The surface was washed with purified water and dried under nitrogen three times.

We imaged mixtures of higher order structures and monomers by AFM. Three sets of sample preparation conditions were used. In the first set, samples were prepared from native PAGE-purified duplex DNA solutions that had been incubated at 4°C for 12 h with 1 KMgTB buffer. Note that this condition does not involve thermal treatment. Samples for the second set of conditions were heated at 90°C for 5 min and incubated at 50°C for 12 or 72 h. In this second strategy, the precursor synapsable DNA was heated to 90°C, which should not affect the G-quadruplex structure but should affect the duplex region. The third procedure was more involved and was chosen to test if under mild conditions of heating the synapsable DNA fiber formation was improved or resulted in significantly different structures than under the other two conditions tested. Gel-purified complementary strands were annealed in the presence of TMACl to obtain precursor duplex DNA. These duplexes were exchanged into the 1 KMgTB buffer using microcentrifugal filters and then incubated at 30°C for 10 min followed by slow cooling to 4°C at a rate of 0.5°C/min. Fibers formed from this protocol are shown in Figures S1 and S2 in Additional file 1.

In summary, the prepared DNA solutions were incubated at different temperatures prior to deposition on the AFM substrate. In the first and second protocols, DNA samples were prepared to test duplex-mediated synapsable quadruplex formation. In many cases, the same stock solutions, or the same samples used for native PAGE, were used for AFM, but they were diluted so that the final DNA concentration applied to the silicon wafer was 1.6 × 10^−4^ kg m^−3^ (0.16 ng/μL). Images were collected in air in tapping mode.

To calculate the average height of the fiber, a trajectory along the fiber was traced to obtain cross sections of the images. This method gives the values of heights along the trajectory of the fiber. A number of points, *N*, were obtained for the fibers in the image being analyzed, and the average and standard deviation of these values were calculated. One fiber representative of those found in each image was used and the value reported. In general, there was a height distribution between fibers and also within each fiber depending on the direction of the cross section. Nevertheless, the distribution was tight (within 1 to 2 nm of the total height depending on the sample). An explanation of the factors that created height variability will be discussed further below. One of those fibers was selected per method of preparation to be reported here.

Persistence length [32] was calculated using a freeware program developed by S. Minko and Y. Roiter. The program calculates persistence length from microscopy images of DNA according to Frontali et al. [33]. The mean is reported along with one standard deviation. For the shortest fibers, eight images were analyzed with a total number of fibers measured equal to 26. In two images, a persistence length (about 600 nm) was obtained. This persistence length was more than one standard deviation away from the average of 203 nm and was not used in calculating the final average and standard deviation. For the longer fibers, six images were analyzed for a total of 30 fibers.

## Results and discussion

### Duplex precursors form synapsable DNA nanofibers

**Figure 1:**
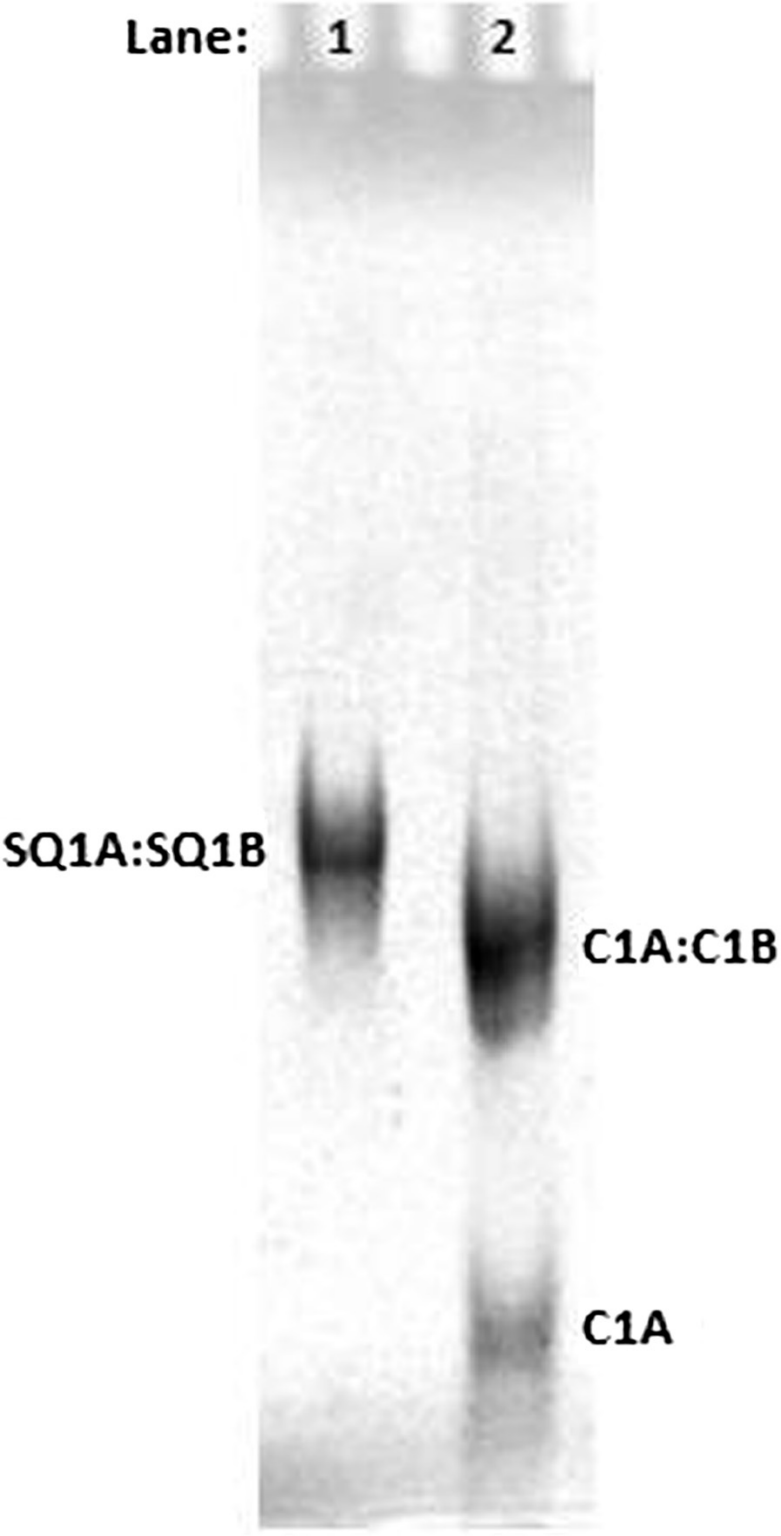
**Duplex precursor assembly in TMACl assessed by native PAGE.** Lane 1, 4.0 × 10^−5^ mol/L (40 μM) SQ1A:SQ1B duplex; lane 2, mixture of 4.0 × 10^−5^ mol/L (40 μM) C1A:C1B duplex and 8.0 × 10^−5^ mol/L (80 μM) single-stranded C1A. C1A:C1B is a 39-mer blunt-end duplex used as a control. SQ1A:SQ1B is the 39-mer synapsable duplex with overhangs. Gel with a mass fraction of 12% acrylamide was run in 0.01 TMgTB buffer and imaged by UV shadowing.

Single-stranded DNA sequences (Table 1) were annealed in TMACl-containing buffer (0.01 TMgTB). The resulting duplexes were purified by native PAGE using standard methods [31]. Figure 1 shows that the SQ1A:SQ1B duplex runs slightly more slowly than the random sequence, blunt-end C1A:C1B duplex control, which is of the same length (39 bases). The C1A:C1B duplex control was used as a migration standard because it shows reproducible gel mobility that is not affected by the presence of overhangs or secondary structure. This result is reproducible over a dozen replicates.

**Figure 2:**
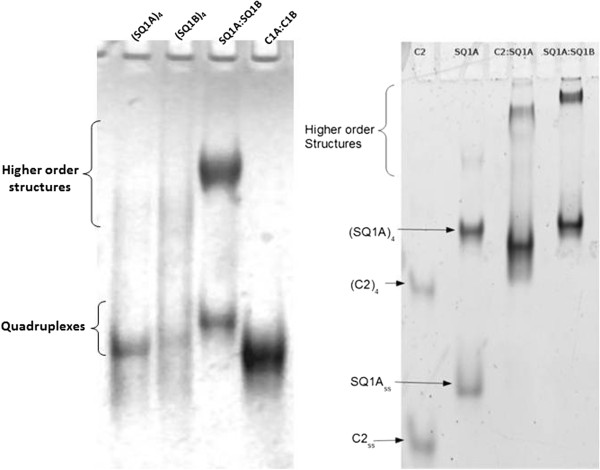
**Native PAGE showing higher order species formed by SQ1A:SQ1B duplex incubated in potassium-containing buffer.** Left: Sample concentrations are 1.0 × 10^−4^ mol/L (100 μM) per strand SQ1A or SQ1B, 5.0 × 10^−5^ mol/L (50 μM) SQ1A:SQ1B duplex, and 5.0 × 10^−5^ mol/L (50 μM) C1A:C1B duplex. Gel (acrylamide mass fraction 12%) was run in 0.01 KMgTB buffer and then UV-shadowed. Right: Sample concentrations are 2.0 × 10^−6^ mol/L (2 μM) strand C2, 2.0 × 10^−6^ mol/L (2 μM) strand SQ1A, 1.0 × 10^−6^ mol/L (1 μM) duplex C2:SQ1A, and 1.0 × 10^−6^ mol/L (1 μM) duplex SQ1A:SQ1B. Gel (acrylamide mass fraction 15%) was run in 0.01 KMgTB buffer and then stained with Sybr Green I dye.

Upon incubation in potassium-containing buffer, the SQ1A:SQ1B duplex assembles into a ‘synapsed’ quadruplex, (SQ1A:SQ1B)_2_. In addition to observation of the (SQ1A:SQ1B)_2_ quadruplex, a much slower mobility species is also observed (Figure 2, higher order structures). These slower migrating species form at the high duplex concentrations used in the UV-shadowing gel experiments (Figure 2, left) as well as in SYBR Green-stained gels loaded with lower DNA concentration samples (Figure 2, right). To test if the assembly of larger species is specific to the SQ1A:SQ1B duplex sequence, we used the C2:SQ1A duplex. This duplex is generated by hybridizing C2, a 29-mer complementary strand, to SQ1A, which results in a duplex with a smaller molecular mass and shorter overall length than the SQ1A:SQ1B duplex. As shown in Figure 2, both the SQ1A:SQ1B and SQ1A:C2 duplexes incubated in potassium-containing buffer form species that migrate more slowly in the gel than the 39-mer homoquadruplexes of C2 and SQ1A.

### Higher order species contain quadruplexes

**Figure 3:**
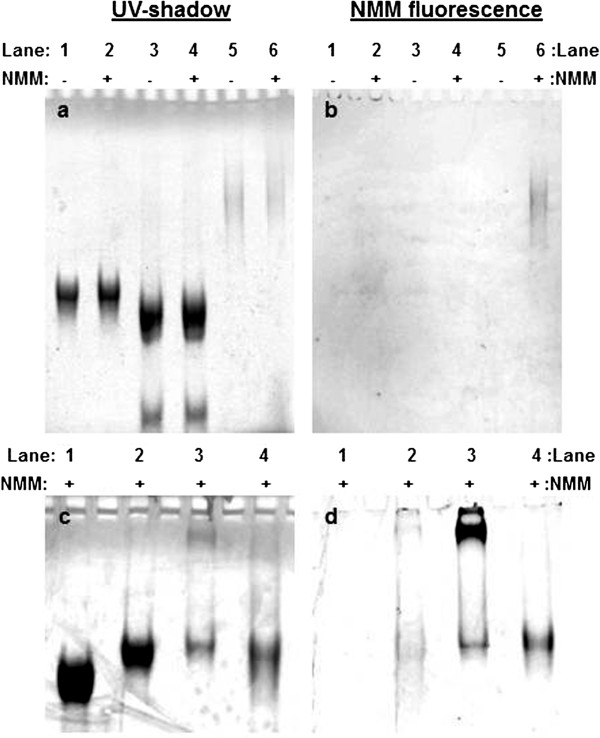
**Native gel electrophoresis images showing that quadruplex is present in synapsed (SQ1A:SQ1B)**_**2**_**.** TMACl (top row): Samples in lanes 2, 4, and 6 contain 1.0 × 10^−5^ mol/L (10 μM) NMM. Lanes 1 and 2, 4.0 × 10^−5^ mol/L (40 μM) SQ1A:SQ1B duplex; lanes 3 and 4, mixture of 4.0 × 10^−5^ mol/L (40 μM) C1A:C1B duplex with 1.0 × 10^−4^ (100 μM) C1A; lanes 5 and 6, 8.0 × 10^−5^ mol/L (80 μM) per strand SQ1A. Gel (acrylamide mass fraction 12%) was run in 0.01 TMgTB buffer and (**a**) UV-shadowed (**b**) or UV-transilluminated. KCl (bottom row): All samples contain 1.0 × 10^−5^ mol/L (10 μM) NMM. Lane 1, 4.0 × 10^−5^ mol/L (40 μM) C1A:C1B duplex; lane 2, 4.0 × 10^−5^ mol/L (40 μM) SQ1A:SQ1B duplex in TMACl; lane 3, 3.0 × 10^−5^ mol/L (30 μM) SQ1A:SQ1B duplex incubated overnight at 4°C in high potassium-containing buffer to assemble quadruplex; lane 4, 6.0 × 10^−5^ mol/L (60 μM) per strand SQ1A. Gel (acrylamide mass fraction 12%) was run in 0.01 KMgTB buffer and (**c**) UV-shadowed or (**d**) UV-transilluminated.

When referenced to the control C1A:C1B duplex, the SQ1A:SQ1B duplex in TMACl (Figure 1) migrates with about the same mobility as the (SQ1A:SQ1B)_2_ quadruplex in KCl (Figure 2). This observation raises the possibility that the bands we ascribe to higher order structures are either simple quadruplexes (i.e., not linked together) or duplexes that link together without quadruplex formation. To test this possibility, gel electrophoresis was performed on samples incubated with NMM, a dye that exhibits increased fluorescence only upon binding quadruplex DNA [34-37]. Figure 3 shows gel images of samples incubated with NMM and analyzed by gel electrophoresis in TMACl (Figure 3a,b) or KCl (Figure 3c,d). Figure 3a shows that incubation of NMM with our samples does not generate new species; a slight shift in band mobility is observed, which is due to NMM binding. Figure 3b,d shows NMM fluorescence intensity recorded for each gel. The control sequence is the preformed SQ1A homoquadruplex, which causes NMM to fluoresce in either buffer (Figure 3b, lane 6; Figure 3d, lane 4). The SQ1A:SQ1B duplex in TMACl does not induce NMM fluorescence (Figure 3b, lane 2), while the synapsed (SQ1A:SQ1B)_2_ quadruplex in KCl clearly does (Figure 3d, lane 3). There is a slight amount of NMM fluorescence for the SQ1A:SQ1B duplex prepared in TMACl and run on the KCl gel (Figure 3d, lane 2), which is an expected result because exposure of the SQ1A:SQ1B duplex to KCl during gel electrophoresis should shift the structure from duplex to quadruplex. The strongest NMM fluorescence is observed for the slowly migrating species formed by (SQ1A:SQ1B)_2_ (Figure 3d, lane 3), indicating that quadruplex is present in this structure.

### Morphology of the synapsable DNA nanofibers by AFM

**Figure 4:**
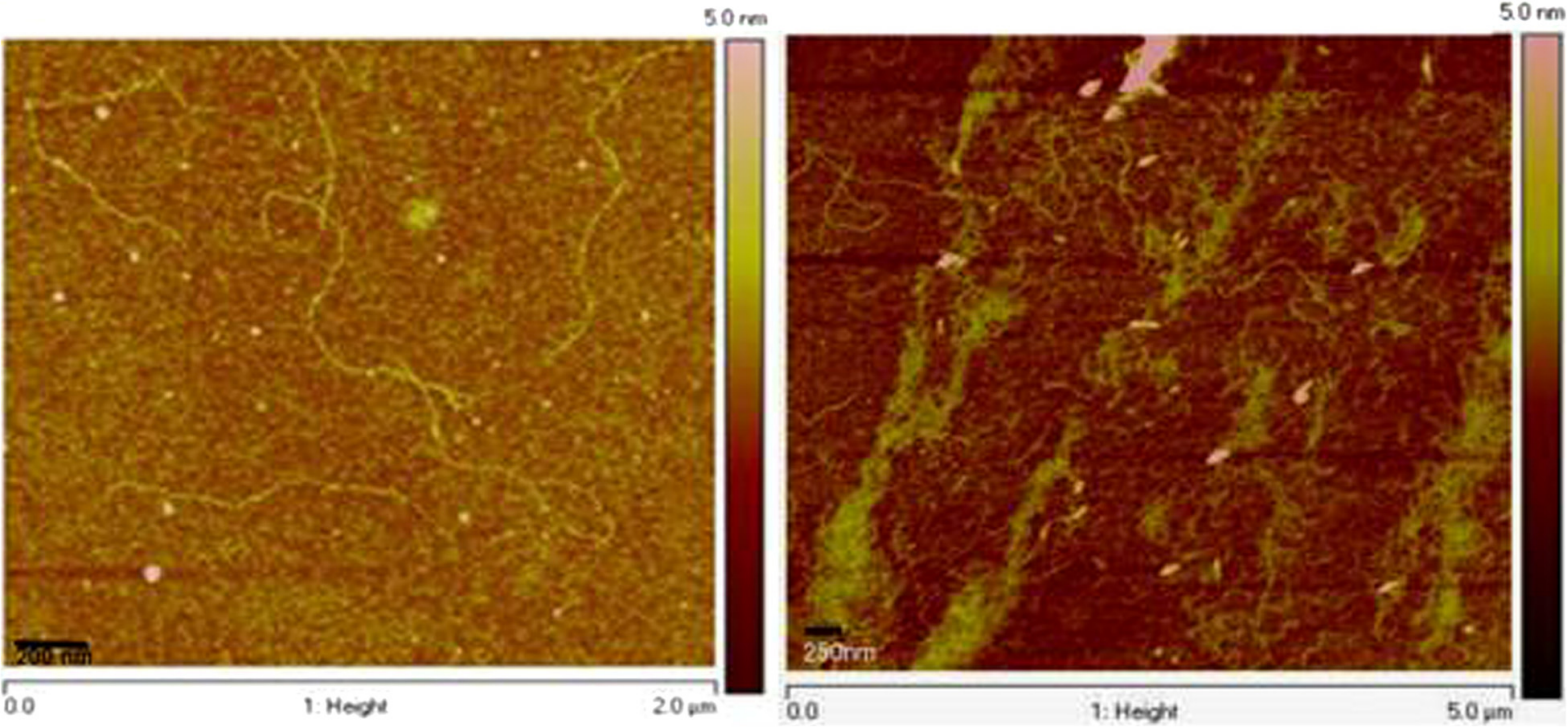
**AFM images of the (SQ1A:SQ1B)**_**2**_**nanofiber.** Left panel: The synapsable DNA nanofiber was prepared by dilution of purified SQ1A:SQ1B duplex originally diluted from 0.05 mol/L (50 mM) TMACl into 1 KMgTB buffer. The quadruplex sample was incubated for 12 h at 4°C prior to depositing it on the silicon wafer for imaging. The average height of the nanofiber is 0.45 ± 0.04 nm. Right panel: Gel-purified SQ1A:SQ1B duplex was heated to 90°C for 5 min and kept at 50°C for 72 h. The concentration was 6.7 × 10^−9^ mol/L (6.7 nM) quadruplex. A drop of sample was placed on the silicon wafer substrate, evaporated for 10 min at room temperature, and then washed with purified water three times prior to drying at room temperature for 1 to 2 h. Average height above the background of the bundles is 0.9 ± 0.4 nm.

On the basis of the gel electrophoresis results indicating that slowly migrating species form quadruplex DNA, we examined solutions of (SQ1A:SQ1B)_2_ using AFM. We observed that fibers form under several conditions with varying morphology depending on the preparation method. Gel-purified duplex DNA precursors formed very long fibers (>2 μm) when incubated at 4°C for 12 h in 1 KMgTB (Figure 4, left). The average height of the nanofiber in Figure 4 is 0.45 ± 0.04 nm. When synapsed samples were heated to 90°C and then incubated at 50°C for 72 h in 1 KMgTB, more fibers were observed by AFM and some of these fibers form bundles with lengths longer than 2 μm (Figure 4, right). The height above the background for these bundles is 0.9 ± 0.4 nm.

The AFM images show that fibers form with lengths ranging from 250 to 2,000 nm and heights from 0.45 to 4.0 nm. The variation in height is most likely due to the existence of the two different regions in the structure: the G-quadruplex box and the duplex arms. G-quadruplexes have a similar diameter to B-form DNA on the basis of AFM measurements [38], although there is a difference in G-quadruplex height depending on whether the quadruplex is unimolecular (1.0 ± 0.2 nm [39] or 1.5 ± 0.3 nm [40]) or tetramolecular (2.2 ± 0.2 nm [39,41]). In our final suprastructures, the duplex arms could be stacked on one another, which could explain the considerable height variation because duplex DNA height depends on the thickness of the hydration layer [38]. Up to a 0.6-nm increase can be observed as a function of hydration [38]. Figures S1 and S2 in Additional file 1 show the existence of at least two height distributions, which are likely due to G-quadruplex and duplex arm regions. We estimate a persistence length, depending on the treatment, that ranges from 161 ± 20 nm for the longest fibers (i.e., Figure 4, left panel). For the shortest fibers, the average persistence length is 203 ± 70 nm, which is within error of the persistence length of the longest fibers. We consistently observe a long persistence length in our fibers, suggesting that this reflects the stiffness of our nanofibers.

Previously, duplex DNA containing a mismatched G-box region has been used to form an unusual G-quadruplex termed ‘synapsable DNA.’ These G-quadruplexes are assembled from duplex precursors and therefore contain two pairs of antiparallel strands. This is unusual as, typically, intermolecular G-quadruplexes containing four separate strands of DNA tend to adopt a parallel strand alignment [42]. The unique structural features of the synapsed quadruplexes have led to the suggestion that they are suitable for building nanostructures [26]. Actual preparation of nanostructures using this strategy has not been demonstrated, however.

**Figure 5:**
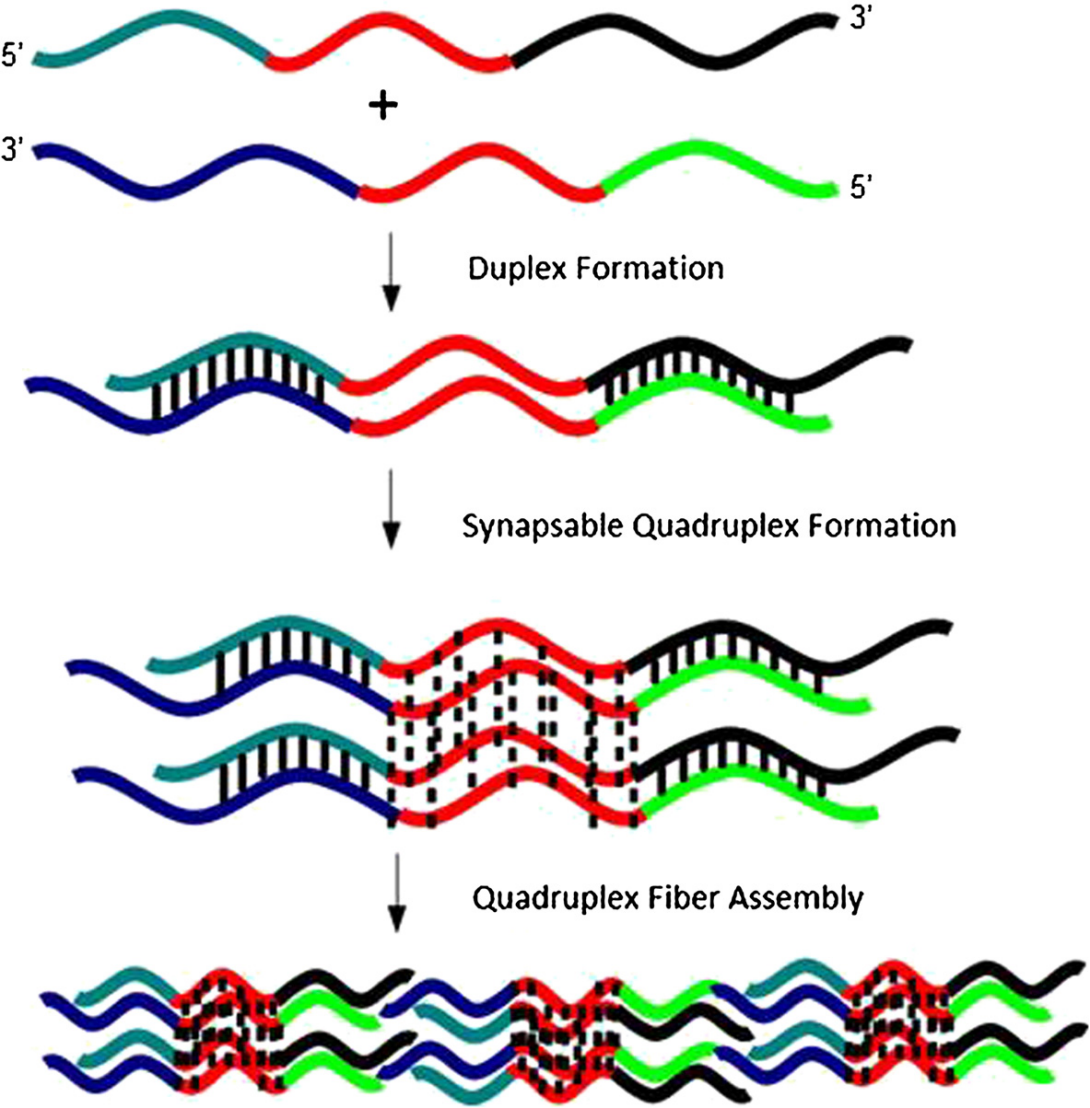
Proposed model for assembly of quadruplex nanofibers.

We aimed to exploit synapsable quadruplex DNA to create a novel, addressable material by adding additional Watson-Crick base pairing regions the synapsable G-quadruplexes. The ‘duplex’ precursor DNA in our design includes a long sequence of guanines in each strand, sequences flanking the G-rich region that are complementary to another strand, and single-stranded overhangs. Formation of the duplex precursor in buffers containing TMACl, which does not facilitate quadruplex formation [43], is observed clearly and reproducibly in our experiments using 0.01 TMgTB. When two duplex precursors associate upon addition of potassium, the final guanine quadruplex contains four DNA strands: two strands are oriented 5′ to 3′ and the other two oriented from 3′ to 5′ (Figure 5). The synapsed quadruplex is assigned using gel electrophoresis on the basis of comparison to control sequences and through quadruplex-specific dye staining experiments. We note that there are several duplex arrangements possible as a result of the orientations in which the duplex precursors can come together. In our design, each synapsed quadruplex contains four duplex ‘arms’ flanking the G-rich region, and each arm has a short single-stranded overhang. To explain fiber formation, we propose that the duplex regions in the quadruplexes partially melt, thereby allowing linking of synapsed quadruplexes together into a larger structure.

Our tentative model for association of (SQ1A:SQ1B)_2_ quadruplexes into fibers involves partial duplex melting, which allows individual quadruplex units to associate into larger fibers (Figure 5). The G-quadruplex region, which contains eight guanines, does not melt at the salt concentrations used in our work [24,27]. After the duplex is incubated in potassium to form a quadruplex, a considerable amount of crowding is introduced at the ends of each G-quadruplex. Under these conditions, it might be more favorable for a (partially) melted duplex region to base pair with a complementary strand in another synapsed quadruplex. Because four strands are available at each end of the G-quadruplex region, the likelihood of occurrence of a single event (base pairing with a strand in another synapsable quadruplex unit) is greatly increased. We observed by AFM that increasing the annealing temperature increases fiber formation, which is consistent with our assembly model. The increased annealing temperature melts the duplex regions more completely, thereby increasing the likelihood that two arms on separate synapsed quadruplex molecules will pair. This model allows for formation of branched structures. This working hypothesis is currently under investigation in our laboratories to test its validity.

Our work is one of the first in which a macromolecular structure is assembled actively via cooperation of Hoogsteen and Watson-Crick base pairing [12]. In contrast, structures such as G4-DNA [44-48], frayed wires [49-51], and G-wires [46] are driven only by Hoogsteen hydrogen bonding in G-quartets. Canonical base pairing has been used to create duplex DNA branches on the ends of frayed wires [49], but initial assembly of the frayed wires exploits only Hoogsteen hydrogen bonding and used a single DNA sequence, which does not allow significant variability/flexibility [49]. Finally, structures created by acid-dependent assembly of d(CGG)_4_ also depend mainly on Hoogsteen hydrogen bonding [52]. In contrast, all of the main DNA fabrication methods using DNA tiles/origami rely on canonical base pairing, with the exception of a structure in which building blocks are connected by quadruplexes rather than duplexes [12]. The presence of duplex and quadruplex elements in our final structures results in distinct recognition sites for incorporation of additional elements [53]. Future work will measure the accessibility and selectivity of these addressable sites in both precursor units and final structures.

## Conclusions

We present a novel strategy to generate fibers with morphologies that differ from duplex-only-based wires. Our method uses hybridization of DNA strands to form duplexes followed by cation-mediated assembly of quadruplexes. The dimensions and quantities of our fibers vary depending on the preparation conditions, but the final assemblies contain quadruplexes. We have shown here the proof of concept for mixed duplex-quadruplex fiber fabrication that we believe holds promise for organized control of fiber assembly.

## Abbreviations

AFM: Atomic force microscopy; NMM: *N*-methylmesoporphyrin IX; PAGE: Polyacrylamide gel electrophoresis; TMACl: Tetramethylammonium chloride.

## Competing interests

The authors declare that they have no competing interests.

## Authors’ contributions

MAM designed the sequences, carried out the gel electrophoresis and AFM measurements, and wrote initial drafts of the manuscript. VAS conducted gel electrophoresis experiments, supervised the design and completion of the work, and wrote the final version of the manuscript. Both authors read and approved the final manuscript.

## Authors’ information

VAS is a project leader in the CNST Energy Research Group. She received an A.B. in Chemistry from Bryn Mawr College and a Ph.D. in inorganic chemistry from Yale University, where her thesis work centered on biophysical measurements of water oxidation chemistry in photosynthesis. After completing post-doctoral work at the University of North Carolina at Chapel Hill, VAS moved to the Department of Chemistry and Biochemistry at the University of Maryland, Baltimore County, where she advanced to the rank of associate professor with tenure. During that time, she and her group elucidated the biophysical chemistry of copper in Alzheimer's disease fibrils and developed methods to create quadruplex-based DNA nanomaterials. VAS joined the CNST in 2010 and is leading projects focused on nanofabrication tools based on biomacromolecular nanomaterials and fundamental measurements of nanostructured catalysts for solar fuels applications.

MAM obtained his Ph.D. degree in chemistry in 2010 working with VAS at the University of Maryland, Baltimore County. Currently, he is an assistant professor and researcher at the School of Medicine and the School of Sciences and Engineering, Politecnico at Universidad San Francisco de Quito. He is a member of GETNano, an Ecuadorian group performing experimental and theoretical research on nanosystems. His research focuses on computational simulations of the self-assembly mechanisms of biomacromolecule-based nanomaterials, evaluation of the suitability of computational methods for modeling non-canonical DNA structures, and development of biosensors based on the nanosystems under investigation in his laboratory.

## Supplementary Material

Additional file 1 PDF document containing buffer formulations and abbreviations, tapping mode AFM images of duplex-quadruplex nanofibers, and a gel electrophoresis image of a control duplex with overhangs. Click here for file
