# Supplementary material for: Synapsable quadruplex-mediated fibers
Source: Nanoscale Res Lett. 2013 May 3;8(1):210. doi: 10.1186/1556-276X-8-210 (PMC3655031; doi:10.1186/1556-276X-8-210)
Supplement: Additional file 1 — PDF document containing buffer formulations and abbreviations, tapping mode AFM images of duplex-quadruplex nanofibers, and a gel electrophoresis image of a control duplex with overhangs. [file 1556-276X-8-210-S1.doc]

**Additional Information File 1**

For “Synapsable-quadruplex mediated G-fibers” by Mendez & Szalai

Table S1: List of buffers used for experiments

| Buffer Abbreviation | Composition | Use |
| --- | --- | --- |
| 0.01-KMgTB | 1.0 × 10^-2^ mol/L (10 mM) KCl, 1.0 × 10^-3^ mol/L (1.0 mM) MgCl_2_, 0.05 mol/L (50 mM) Tris-borate, pH 8.0 | Native gel electrophoresis |
| 0.01-TMgTB | 1.0 × 10^-2^ mol/L (10 mM) TMACl, 1.0 × 10^-3^ mol/L (1.0 mM) MgCl_2_, 0.05 mol/L (50 mM) Tris-borate, pH 8.0 | Duplex precursor preparation; native gel electrophoresis |
| 1-KMgTB | 1.0 mol/L (1 M) KCl, 1.0 × 10^-3^ mol/L (1.0 mM) MgCl_2_, 0.05 mol/L (50 mM) Tris-borate, pH 8.0 | Synapsed quadruplex preparation |

.


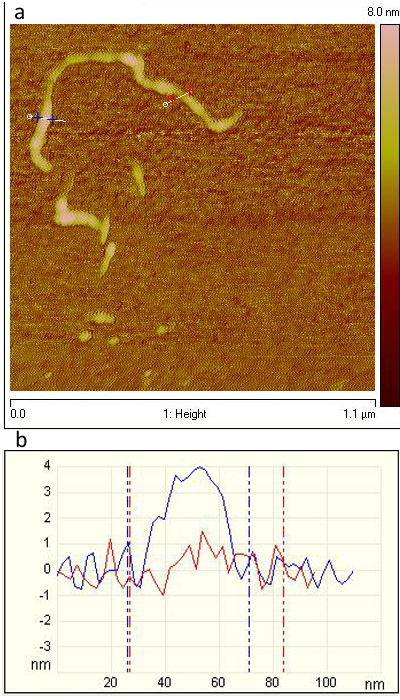

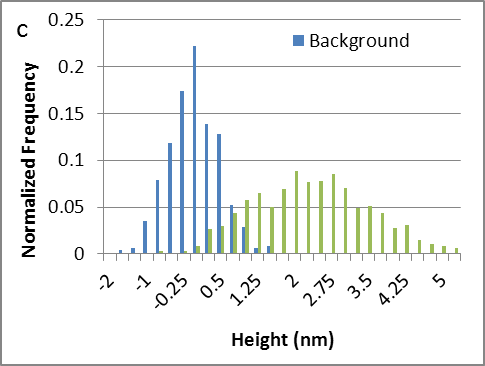


**Figure S1.** Tapping mode AFM image (a) of fiber formed by (SQ1A:SQ1B)_2_ after heating the sample to 30 °C and slowly cooling it to 4 °C. (b) Profile showing that this fiber has at least two height regions. The heights of the two regions are estimated to be 1.6 nm ± 0.2 nm and 3.2 nm ± 0.4 nm height over the background. The fiber was prepared from duplex prepared in TMACl and exchanged into K^+^ buffer. Duplex was prepared from gel-purified single-stranded oligonucleotides, but was not purified by PAGE after duplex formation. (c) Normalized histogram of heights for background (N=483) and nanofiber (N=679).


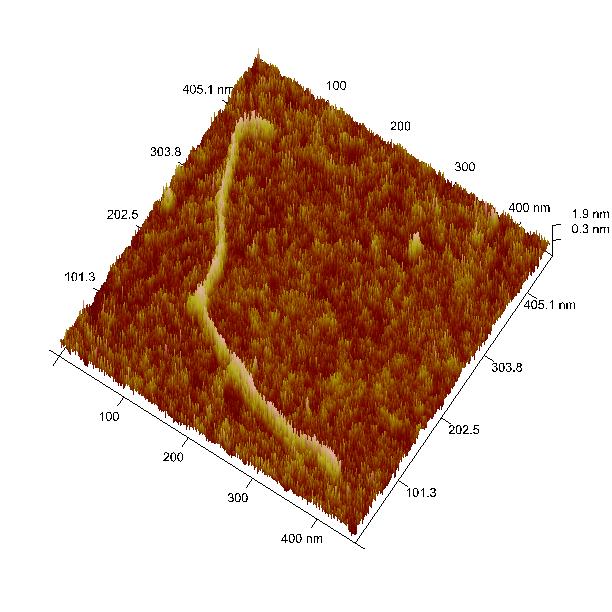


**Figure S2.** 3D tapping mode AFM image of fiber formed by (SQ1A:SQ1B)_2_ after heating the sample to 30 °C and slowly cooling it to 4 °C. The average fiber height is 1.4 nm ± 0.6 nm. The fiber was prepared from gel purified SQ1A and SQ1B oligonucleotides.


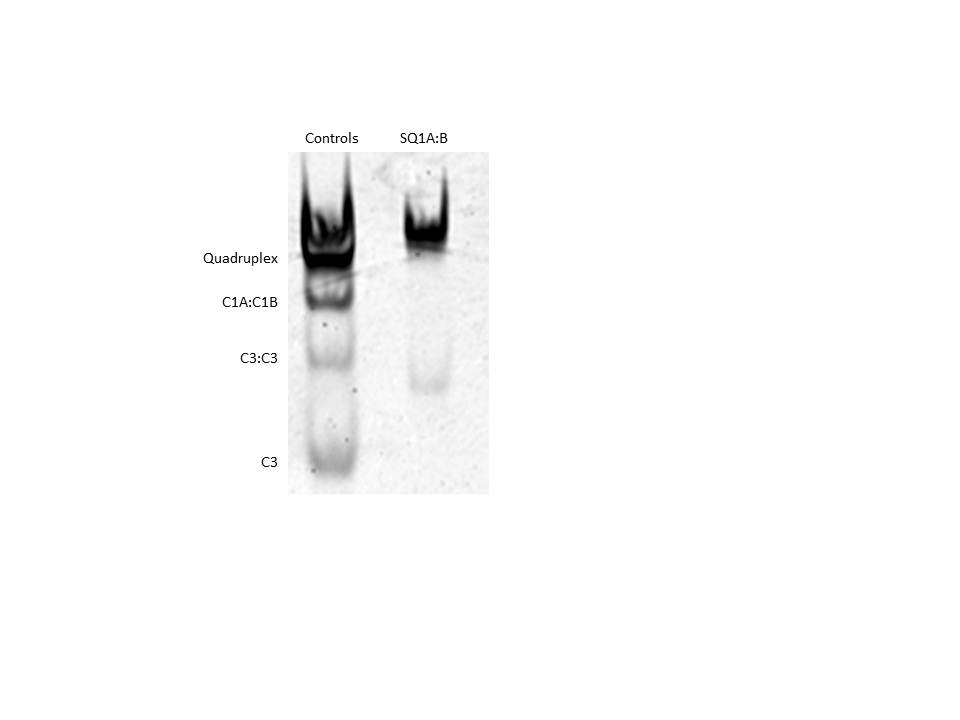


**Figure S3**: Native PAGE (acrylamide mass fraction 12%) of samples prepared in 1-KMgTB buffer. The gel shows bands for four control sequences: a 24-mer single stranded oligonucleotide (C3), its self-complementary duplex (C3:C3), the 39-mer duplex (C1A:C1B) and a 39-mer quadruplex DNA with overhangs. C3 is a self-complementary sequence derived from the thrombin binding aptamer that includes 10-base overhangs, 5'-dGGT TGG TGT GGT TGG AAT TCC AAC-3' The 39-mer quadruplex sequence was 5'-dACA GTA GAG ATG CTG CTG AGG GGG GGG TGT GCT TCA AGC-3'. The SQ1A:B duplex, which forms quadruplex under these conditions, also was run on the gel.
